# Self-Portrait with the Spanish Flu

**DOI:** 10.1007/s10393-019-01466-8

**Published:** 2020-02-29

**Authors:** Mark Olival-Bartley

**Affiliations:** Munich, Germany

Self-portrait with the Spanish Flu

The scream evokes the dream I dread.

“I want to sit up,” you had said,

regarding the chair.

I carried you there

from the bed.

Tears were shed

but not by you. You drew cold air

and shut your eyes, ever the fair

sister I adore.

The halos of your

backlit hair

led my stare

from their gilt sheaves to the cracked score

of the window-panes’ ferny hoar

frost. Your soul, wrested

from its maidenhead,

found the door.

Metaphor.

This phthisis has disquieted

the Muses and dispirited,

yea, Apollinaire,

his devil-may-care

and hurt head,

shepherded

to futter the fields of trouvère.

Endless black weddings fill the air

with song and implore

the dispatch of war-

like despair;

everywhere,

we fall sick with fevers that pour

gloom into us and dull the core

of bright hues that fed

on what painters bled

from a door

heretofore.

Mark Olival-Bartley

## About the Poem and the Poet

Comprising six syllabic sestets of a Welsh form called clogyrnach, this ekphrastic poem is a dramatic monologue that imagines the mindset of Edvard Munch in his *Self*-*Portrait with the Spanish Flu*, which was painted in 1919 when the artist was fifty-six years old. The first three stanzas allude to a lifelong trauma caused by witnessing the tubercular death of Sophie, his favorite sibling; the chair depicted is the same wherein she died, which, incidentally, is on view at Oslo’s Munch Museum. Having survived injuries sustained in the First World War, surrealist poet Guillaume Apollinaire contracted the influenza A virus, subtype H1N1, and died in November 1918 at the age of thirty-eight. A black wedding—often referred to by its Yiddish term, *shvartze khasene*—was an ancient Jewish rite to ward off an infectious epidemic through a community-funded marriage ceremony between two indigent strangers that took place in a cemetery.

Mark Olival-Bartley is the poet in residence at EcoHealth Alliance. He tutors composition, creative writing, and American literature at LMU Munich, where he is presently writing his dissertation on E. A. Robinson’s metasonnet.

## About the Art and the Artist

Edvard Munch contracted the Spanish flu at the end of 1918 and documented the illness in a series of sketches and paintings. With a sallow complexion and thinning hair, Munch is sitting in a wicker chair in front of his unmade bed, illustrating his frail condition. The figure is depicted using simple lines and minimal colors applied with rough sweeps of the brush. The small room evokes a feeling of confinement, and the dominant use of yellow intensifies the restlessness of the composition.

Edvard Munch was born in 1863, in Löten, Norway, and was a key artist of the Expressionism movement. Closely associated with Symbolism, he is best known for his images of isolation, anxiety, sensuality, rejection and death, many of which reflected his neurotic and tragic life.

